# Numerical and Physical Study on New Simple Design of Subflux Flow Controller for One-Strand Tundish

**DOI:** 10.3390/ma15113756

**Published:** 2022-05-24

**Authors:** Adam Cwudziński

**Affiliations:** Department of Metallurgy and Metals Technology, Faculty of Production Engineering and Materials Technology, Czestochowa University of Technology, Armii Krajowej 19, 42-201 Czestochowa, Poland; kmitm@wip.pcz.pl or adam.cwudzinski@pcz.pl

**Keywords:** tundish, subflux flow controller, hydrodynamics, numerical simulations, water model

## Abstract

Tundish metallurgy is essential for continuous steel casting technology. In this study, the subflux flow controller (SFC) installed in the tundish pouring zone was tested, demonstrating the possibility of simultaneously reducing the dimensions of the flow control device (FCD) and effectively influencing the structure of the liquid steel flow. On the basis of computer simulations and water model trials, results were obtained describing the hydrodynamic structure in considered variants of the one strand slab tundish. Considering the influence of the SFC on the steel flow structure in the tundish, and the gradient of the wall shear stress and total pressure on the SFC surface/tundish walls, the most optimal SFC variant for a single-strand wedge-type tundish is SFC No. 2C.

## 1. Introduction

One of the branches of extractive metallurgy is steel pyrometallurgy. The manufacturing of steel includes smelting, secondary metallurgy and casting processes. Continuous casting is the dominant method of producing semi-finished steel products at present. During the process, the molten steel being poured from the ladle to the mold flows through the tundish. The tundish enables multiple heats to be cast successively in the same casting sequence. Therefore, the wear of the tundish and its effect on the movement of the molten steel are essential for continuous casting technology. Hence, the tundish’s metallurgy is a fundamental area for continuous casting of billets, blooms or slabs. Scientific studies in the field of the tundish include the following issues related to the refining of liquid steel [1,2,3,4,5,6]: the behavior of the tundish powder [7,8,9,10,11,12], reactions in the non-metallic inclusions-steel-slag system [13,14,15,16,17], steel micro-alloying [18,19,20], refractory lining wear [21,22,23,24], or modification of the hydrodynamic structure in the working space of the tundish [25,26,27,28,29,30]. Physical and numerical models are used in the search for new technological solutions to optimize the process of continuous casting of steel in the context of the tundish. For physical modeling, water models which meet the similarity criterion of Froude (Fr) or Fr and Reynolds (Re), are still successfully used. Although, in the context of multiphase flows and meeting the simultaneous similarity criteria of Re, Fr and Weber (We), the use of tin as a model liquid is the most appropriate [31]. On the other hand, the vast majority of the numerical models are based on the averaged Navier-Stokes equation and two-equation models of turbulence, representing the chaotic motion of liquid steel. In the context of numerical models, studies that optimize the models themselves are, of course, carried out in order to obtain results that are closest to real industrial processes [32,33]. The tundish should ensure a stable flow of molten steel to the mold throughout the casting sequence. Therefore, the working space of the tundish should generate a hydrodynamic structure with the smallest possible share of stagnant flow, minimizing the erosive action of molten steel on the refractory lining and ensuring stable behavior of the tundish powder. Most of the tundishes used in industrial conditions are equipped with FCD: dams, weirs, turbulence inhibitors, gas-permeable barriers. The FCD is made of refractory materials with a specific erosive strength, which is influenced by temperature and pressure in the system, and hydrodynamic structures. Therefore, in order to reduce the additional sources contributing to the generation of exogenous non-metallic inclusions (NMIs), solutions that optimize the flow of liquid steel are tested, based on the use of electromagnetic mixers or advanced ladle shrouds [34,35,36,37,38,39]. In this study, the subflux flow controller (SFC) installed in the tundish pouring zone was tested, demonstrating the possibility of simultaneously reducing the dimensions of the FCD and effectively influencing the structure of the liquid steel flow.

## 2. Tundish and Flow Control Devices

**Figure 1:**
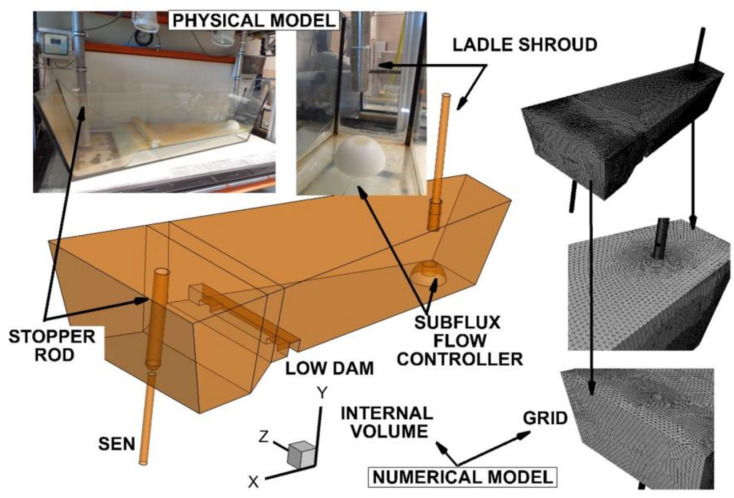
Numerical and physical model of tundish with subflux flow controller.

For slabs casting, a tundish in the shape of a wedge, filled with steel up to the level of 22 tons, was designed (Figure 1). A detailed description of the tundish is presented in the work [40]. As part of the studies, a flow control device, installed in the tundish’s pouring zone, was tested. An SFC with an outer hemisphere shape was tested with, and without, a cavity (Figure 1). The longitudinal axis of the cavity coincides with the longitudinal axis of the ladle shroud. SFCs with hemisphere base diameters of 0.2 m, 0.3 m, 0.4 m and 0.5 m were tested. The basis of SFC engineering design is the simultaneous reduction of additional refractory material being introduced into the working space of the tundish (minimum diameter of the base) and increasing resistance of the device to mechanical wear (streamlined shape), while effectively influencing the hydrodynamics of steel flow during casting (the cavity in the central axis of the SFC) [41]. The immersion depth of the ladle shroud, with an internal diameter of 0.07 m in all of the considered variants of the tundish, was 0.25 m. Casting simulations were made for a 1.5 m × 0.225 m slab. In order to assess the stability of the proposed flow control device, different casting speeds and degrees of overheating of the liquid steel were tested (Table 1). The bare tundish was only equipped with a low dam. 

## 3. Methodology

The mathematical and physical model is described in detail in works [40,42]. Computer simulations were performed in Ansys-Fluent 12.1. The computational grid of the tundish model was built of tetrahedral elements of the t-grid type Tet/hybrid, with an average number of 690,000 elements. The quality of computational grid was checked by angle of skewness and all models fulfilled requirements for sufficient mesh quality in accordance with the methodology presented in the author’s previous works [40,43]. A pressure-based solver was used and calculations for mass, momentum and energy transfer were performed under steady-state conditions. Then, the distribution of the marker in the working space of the tundish, as a function of time, was simulated, registering change in its concentration (transient simulations). The transport simulation of the marker for the transient conditions was conducted with the species model, taking into account the properties of the liquid steel and the marker. The realizable k-epsilon model was used to describe the turbulence. In the tundish’s wall zone, the standard wall function with y+ parameter between 30–60 was used. The free surface of liquid steel was simulated with the boundary condition of a wall with zero tangential stresses. For simulations of slab casing with 0.9 m/min, the liquid steel enters the tundish at a velocity of 1.316 m/s. Additionally, turbulence intensity of the liquid steel inflow into tundish was assumed to have a turbulence kinetic energy of 0.0173 m^2^/s^2^ and dissipation rate of 0.065137 m^2^/s^3^. Properties of liquid steel were presented in the author’s previous work [40]. The author’s previous work on general liquid steel properties and steel flow were compared with industry liquid steel behavior with quite good conformity [44]. In this work, liquid steel grade was characterized by average content of carbon 0.16 wt.%, manganese 1.36 wt.% and silicon 0.26 wt.%. The behavior of non-metallic inclusions was simulated with the discrete phase model (DPM) with turbulent transport functions (Random Walk Model and Random Eddy Lifetime). The transport of non-metallic inclusions was coupled with the forces of buoyancy, drag, lift, virtual mass force and pressure. The DPM model was clearly described in detail in [45,46,47]. Non-metallic inclusions could flow out of the tundish through a tundish nozzle with molten steel into the mold, or be removed from the steel after contacting a free surface. Of course, in industrial conditions, mere contact of the NMIs with the free surface covered with the slag phase does not guarantee effective refining of steel from inclusions; therefore, the obtained results indicate only flotation conditions. A group of 5000 NMIs of types Al_2_O_3_ and CaO, with densities of 3960 kg/m^3^ and 3340 kg/m^3^, respectively, were simulated. The NMIs’ motion was simulated with a spherical shape, having a diameter of 5 µm. The size of NMI was chosen on the basis of the author’s previous work, where the majority of the largest NMIs detected in the tundish had a diameter ranging between 4 and 6 μm [48]. For discretization, the Green-Gause Cell Based concept for spatial gradient was used. Moreover, for pressure standard and for momentum, energy and turbulence transport equations, the second order upwind procedure was used. A physical glass tundish model, equipped with a system for measuring changes in the electrical conductivity of water, was made on a 0.4 scale. Fulfilling the Froude similarity criterion for steel and water, the casting of slabs in isothermal conditions was simulated. On the scale of 0.4, a model of the flow control device was made using the CNC technique. A 2% NaCl solution was used as a marker during the laboratory experiments where the E-type residence time curve was recorded. The level of NaCl in the solution gives comparable values for tracer and water density. Therefore, influence of tracer density on tracer distribution in the water can be considered negligible. On the basis of the recorded and calculated RTD curves, the shares of stagnancy and active flow were calculated in accordance with the combined model proposed by the author’s works [49,50]. 

## 4. Results and Discussion 

### 4.1. Physical Trials—Water Model

**Figure 2:**
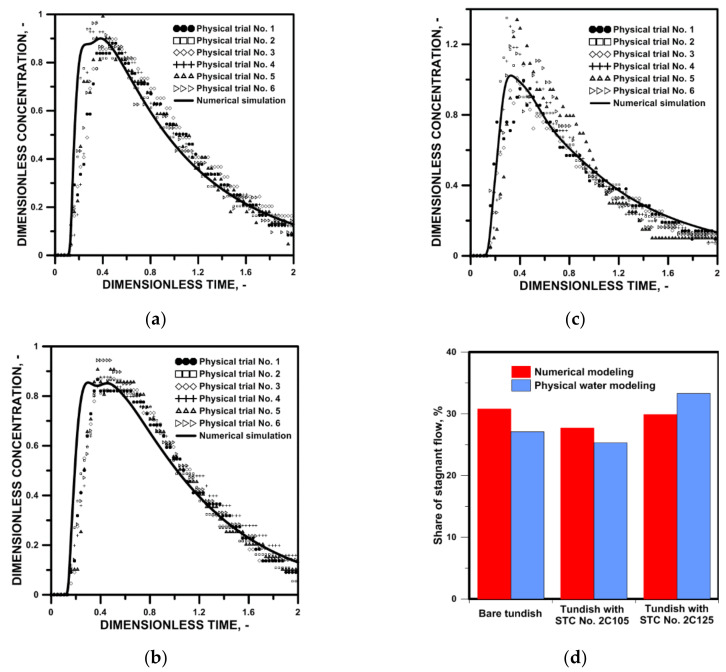
Physical water model trials: (**a**) E-type residence time distribution curve for bare tundish, (**b**) E-type residence time distribution curve for tundish with SFC No. 2C105, (**c**) E-type residence time distribution curve for tundish with SFC No. 2C125, (**d**) comparison of stagnant flow zones.

The first stage of the studies was the validation of the numerical model, in particular, the application of the realizable k-epsilon model describing the phenomena related to turbulent mass and heat transport. Comparing the results obtained during the laboratory experiments with the results from computer simulations gives engineers the opportunity to critically refer to the results obtained. The qualitative distributions of the tracer concentration obtained for isothermal conditions for molten steel (numerical modeling) and water (physical model) showed satisfactory agreement (Figure 2a–c). Times to reach the maximum concentration on the RTD curve for the bare tundish and with SFC No. 2C125, were 0.38 and 0.32, respectively. However, in the variant of the tundish with SFC No.2C125, a lower dispersion of the marker in the aqueous model, in relation to the computer simulation, was observed (Figure 2c). Whereas, in the case of the variant of the installation of the tundish SFC No. 2C105, two peaks in the top zone were revealed on the RTD curve from the computer simulations. On the other hand, the RTD curve for the same tundish installation variant obtained during physical modeling was characterized by one rather flattened peak. Therefore, for the quantitative analysis, on the basis of the obtained RTD curve distributions, the share of the stagnation zone in the tested tundish variants was selected. Slight discrepancies in the percentages between the results of computer simulations and experiments on the water model for stagnant flow, ultimately show the same tendency indicating optimization of the hydrodynamic structure in the tundish equipped with SFC No. 2C105. Ultimately, it can be concluded that, although the realizable k-epsilon model does not perfectly reflect the industrial state, its compliance and ability to predict trends is sufficient for it to be used as an effective tool to optimize the operation of industrial tundish. 

### 4.2. Numerical Simulation

#### 4.2.1. Liquid Steel Behavior in the Considered Tundish Equipment

**Figure 3:**
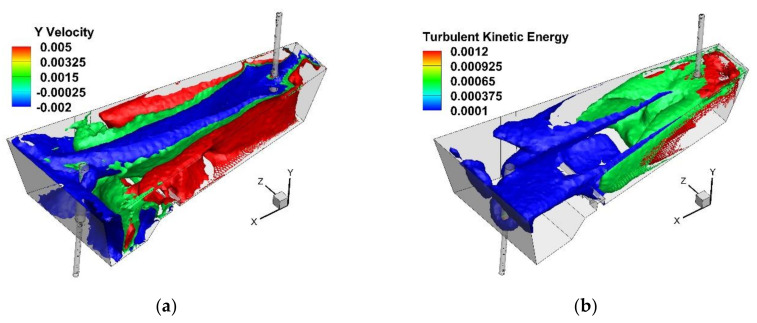
Bare tundish—liquid steel behavior: (**a**) Y component velocity—m/s, (**b**) turbulent kinetic energy—m^2^/s^2^.

On the basis of the computer simulations, results were obtained describing hydrodynamic structure in the considered variants of the tundish. The hydrodynamic condition of a wedge-type single-nozzle tundish equipped only with a low dam is described in detail in the author’s previous works [19,40,42]. Also, in the variant of this tundish considered in this paper, dominant features include a ladle shroud immersed at 0.25 m, and two recirculation structures located on both sides of the longitudinal axis of the tundish, falling down in its central part and rising at the longitudinal side walls (Figure 3a). The liquid steel, after contact with the bottom in the pouring zone, flows towards the longitudinal side walls and then towards the free surface, and then it falls in the central part of the tundish towards the bottom. The effect of vertical recirculation of the feed stream is a characteristic of one-strand tundishes. It can also be seen that in the central part of the tundish the descending stream flows at a speed half the speed of the rising stream in the zones near the wall. The process of the turbulent mass transport in the tundish is the result of the decomposition of the kinetic energy of the turbulence, and when it weakens, the mixing process related to the chemical homogenization of the system declines. In the tundishes without an additional injection of argon, the only driving force for the system is the feed stream and the amount of turbulence kinetic energy (TKE) it carries. In all of the considered variants of tundish installation, the initial turbulence kinetic energy of the feed stream was 0.0173 m^2^/s^2^. Figure 3b shows the distribution of TKEs for the three levels, 0.5%, 3% and 69% of the initial TKE value in the base tundish. The 3% level is the mean TKE value and its spatial distribution is described by the green contour in Figure 3b. Therefore, in a tundish with a low dam, the farther towards the stopper rod system, the less turbulence of the system and the affecting force of the feed flow. 

**Figure 4:**
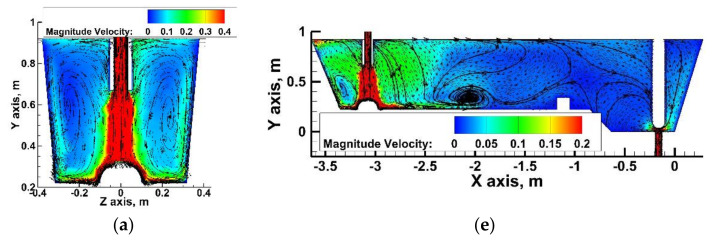

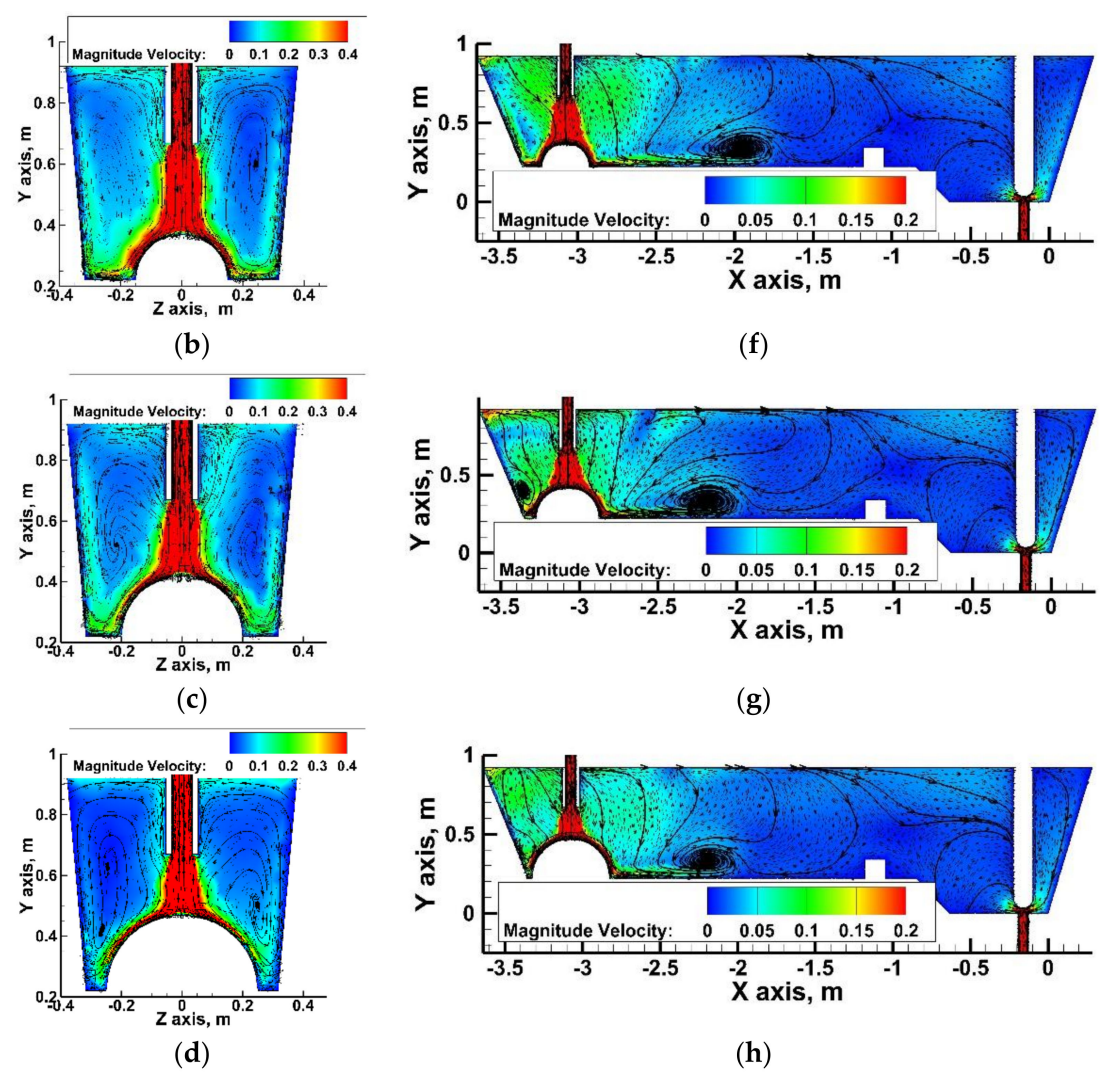
Liquid steel flow in the tundish with hemisphere SFC: (**a**) pouring zone (transversal plane—case No. 2), (**b**) pouring zone (transversal plane—case No. 3), (**c**) pouring zone (transversal plane—case No. 4), (**d**) pouring zone (transversal plane—case No. 5), (**e**) pouring and stopper rod zones (longitudinal plane—case No. 2), (**f**) pouring and stopper rod zones (longitudinal plane—case No. 3), (**g**) pouring and stopper rod zones (longitudinal plane—case No. 4), (**h**) pouring and stopper rod zones (longitudinal plane—case No. 5).

The working space of the tundish was modified with a subflux flow controller mounted in the axis of the tundish feeding stream. In the first study stage, the hemispherical SFC was tested. Figure 4 shows the results of the liquid steel flow and the distribution of the streams on two transverse and parallel planes with respect to the longitudinal axis of the object. The larger the SFC size, the lower the value of the molten steel velocity acting on the tundish’s bottom. The size of the SFC does not significantly affect the behavior of the liquid steel and its directions of circulation in the tundish’s pouring zone. The effect of the SFC on the flow structure in the area between the tundish pouring and emptying zones is also limited. In the center of the tundish, the molten steel jets descend and are forced towards the feed zone by the back streams flow from the stopper rod system zone. With a SFC with a base diameter of 0.4 m and 0.5 m, there is a shift of the liquid steel circulation area at the bottom towards the feed zone, which is related to slowing of the momentum of the feed stream and the dominance of back streams. 

**Figure 5:**
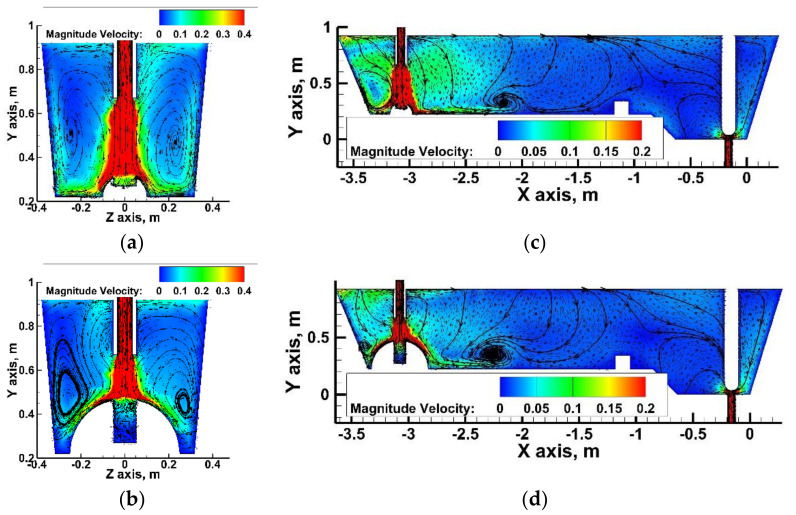
Liquid steel flow in the tundish with modified hemisphere SFC: (**a**) pouring zone (transversal plane—case No. 6), (**b**) pouring zone (transversal plane—case No. 9), (**c**) pouring and stopper rod zones (longitudinal plane—case No. 6), (**d**) pouring and stopper rod zones (longitudinal plane—case No. 9).

The mean flow velocities of liquid steel for the hemispherical SFC tundish variants were 0.038 m/s, 0.036 m/s, 0.035 m/s and 0.034 m/s for SFC No. 1, No.2, No. 3 and No. 4, respectively. Hence, in relation to the flow velocity of the liquid steel in the bare tundish, where the steel flows at an average speed of 0.038 m/s only in the variant of the tundish with SFC No. 4, was there a decrease in the average flow velocity of the liquid steel at the level of 10%. The cavity in the central part of the SFC is filled with liquid steel and forms a buffer between the supply stream and the regulator surface exposed to its direct impact. Increasing the radius of the hemisphere changes the depth of the cavity and, as a result, causes a different recirculation of steel in the cylinder. The streams, circulating in the cavity, interact with the supply stream, slowing down its momentum (Figure 5a,b). However, the use of a cavity in the SFC does not significantly change the macro-nature of the flow of streams forming in the central working space of the tundish (Figure 5c,d). 

**Figure 6:**
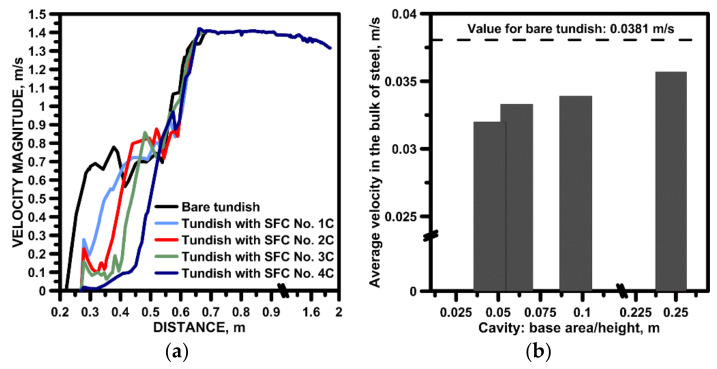
Liquid steel velocity magnitude in the tundish with modified SFC: (**a**) along vertical axis in the tundish pouring zone, (**b**) average velocity in the bulk of steel.

Figure 6 shows the change in the flow velocity of the liquid steel along the axis of the ladle shroud and SFC in the tundish’s feeding zone. In the tundish without SFC, a 50% reduction in speed is noticeable after flowing about 0.1 m from the moment the stream flows out of the ladle shroud. In the bare tundish, oscillating around the velocity value of 0.7 m/s (±0.05 m/s), the stream flows towards the tundish bottom, decelerating only at a height of 0.05 m from the bottom of the tundish, due to interaction with the streams circulating in the tundish supply zone. Compared to the tundish without SFC, the use of a flow regulator reduces the flow rate of the feed stream by 40–85%, depending on the radius of the base of the hemisphere. Additionally, an increase in the radius of the hemisphere by each successive 0.05 m in the tested range results in gradual reduction of the velocity of the liquid steel, by an average of 0.1 m/s, at the point of contact of the supply stream with the free surface of the steel in the cavity. The designed flow control device also influences the steel flow intensity in the entire working space of the tundish (Figure 6b). 

By changing the radius of the hemisphere base, the depth of the cylinder was also changed, keeping the distance between the base of the cylinder and the hemisphere base at 0.05 m. In the SFCs No. 1C-4C the radius of the cylinder base was the same. Therefore, the ratio of the base of the cylinder to its height changed, reaching, for example, the value of 0.045 in the case of SFC No. 4C. In the case of SFC No. 4C the average steel flow velocity in the tundish was reduced by 15% relative to the bare tundish (Figure 6b). 

**Figure 7:**
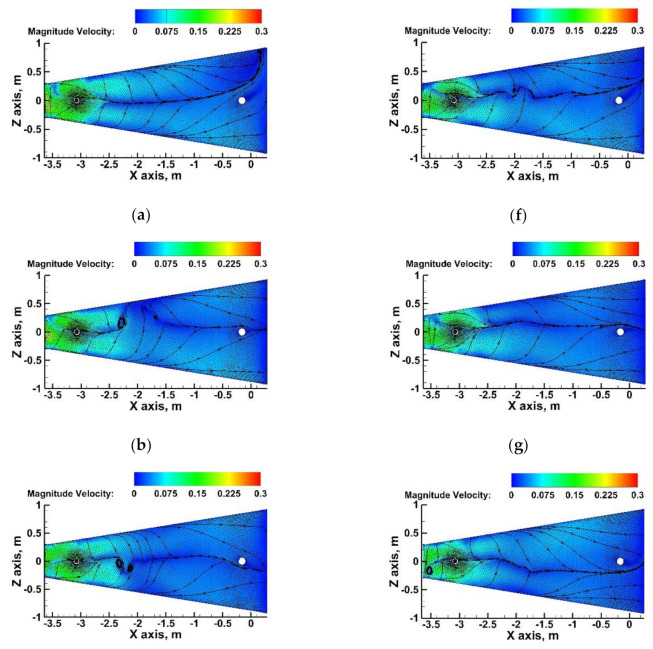

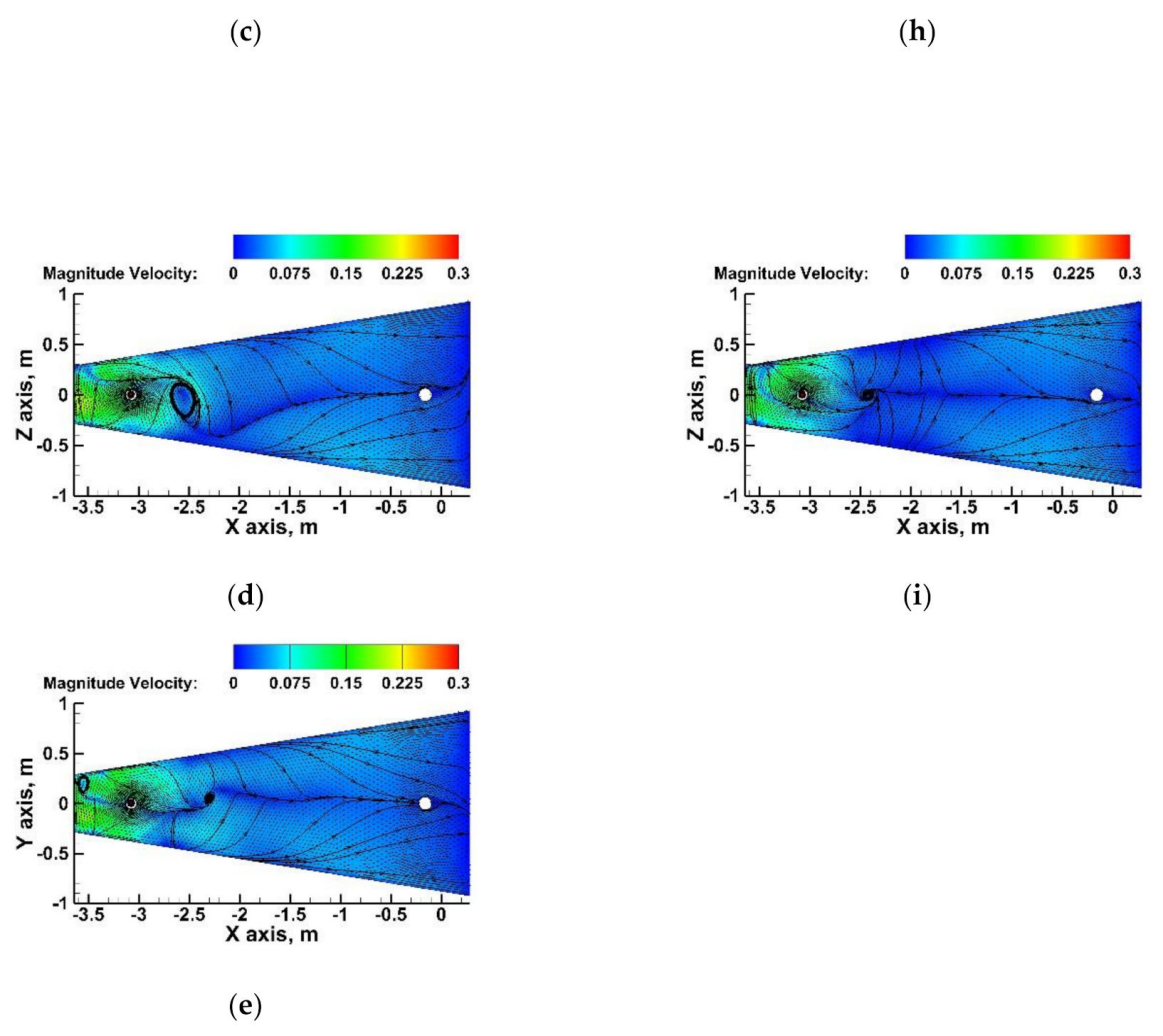
Behavior of liquid steel flow near the tundish flux-steel interface: (**a**) bare tundish, (**b**) tundish with SFC No. 1, (**c**) tundish with SFC No. 2, (**d**) tundish with SFC No. 3, (**e**) tundish with SFC No. 4, (**f**) tundish with SFC No. 1C, (**g**) tundish with SFC No. 2C, (**h**) tundish with SFC No. 3C, (**i**) tundish with SFC No. 4C.

An important issue for tundish metallurgy is the determination of the steel flow velocity at the interface between the metallic and slag phases, in order to assess the possibility of the entrainment of non-metallic phase droplets by steel streams and their transport to its volume. Figure 7 shows the maps of the velocity and direction of liquid steel flow with a limit value of 0.3 m/s; when this is exceeded, it is possible to initiate the phenomenon of transferring the non-metallic phase to the liquid steel [51,52,53]. In none of the variants of the installation of the SFC tundish with, and without, a cavity were velocity areas characterized by exceeding the critical speed value. In the pouring zone of the tundish, velocity values of 0.2 m/s occurred as a result of stimulating the hydrodynamics of the steel movement by the feed stream. Attention is drawn to the directions of the liquid steel flow, which, in this zone of the tundish, revealed local hydrodynamic modifications in the various variants of the tundish’s installation. 

In the case of using the SFC without a cavity in the zone at the surface flow of liquid steel, clear vortex structures and quite significant asymmetry of the flow, in relation to the longitudinal axis of the tundish, were visible (Figure 7b–e). The vortex of the steel flow, and hence the likelihood of the formation of vortex funnels, clearly limits the use of a cavity in the SFC (Figure 7f–i). A more symmetrical flow of the molten steel is also observed with respect to the longitudinal axis of the tundish. In the case of the tundish with SFC No. 4C, the hydrodynamic structure outside the feed zone is the most symmetrical and is additionally modified by backflows located approximately 1 m from the feed zone (Figure 7i). 

#### 4.2.2. Hydrodynamic Conditions in the Considered Tundish

**Figure 8:**
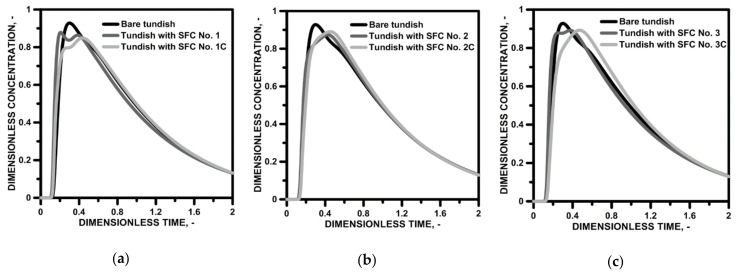

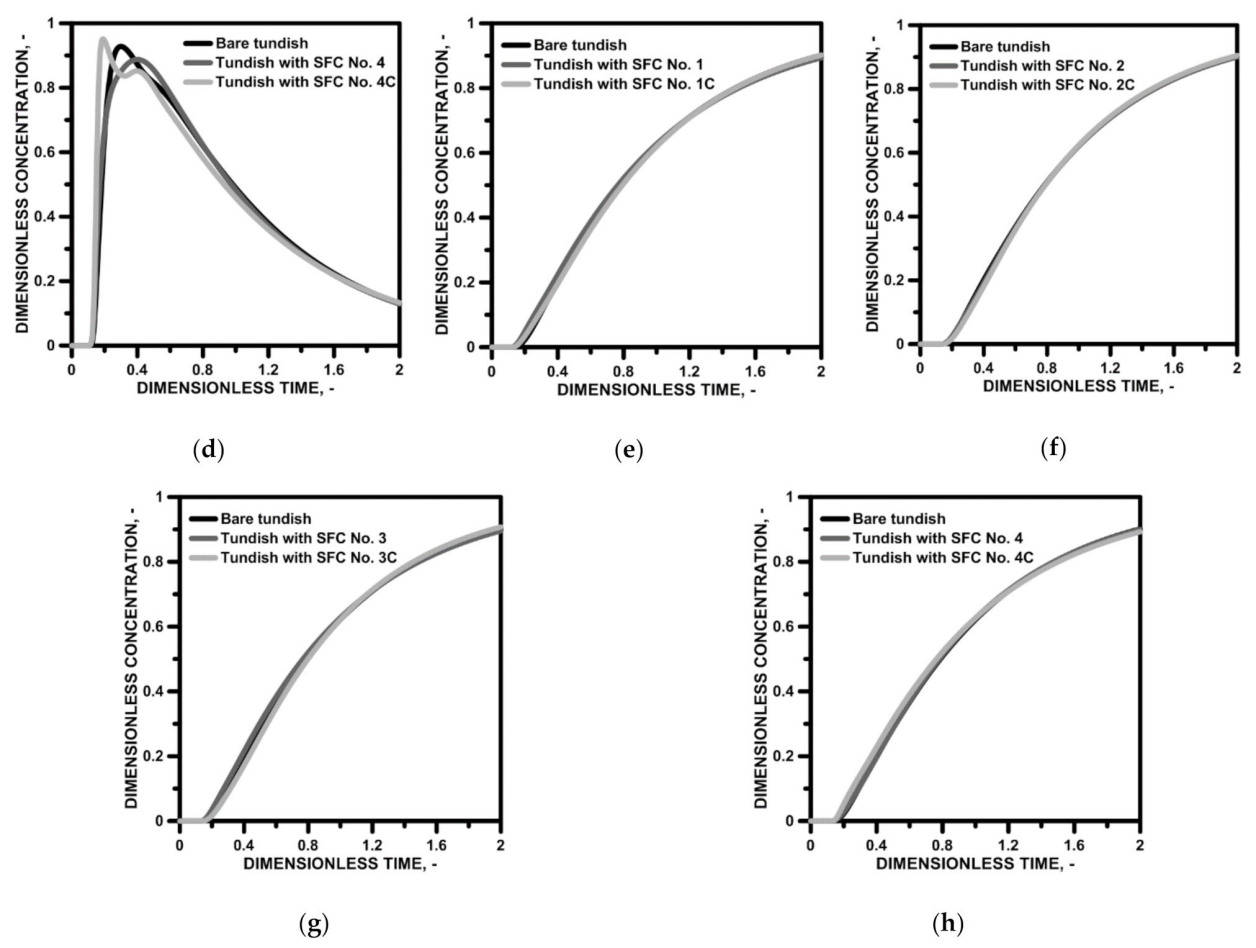
Residence time distribution curves for bare tundish and tundish with SFC: (**a**) E-type curves for SFC No. 1 and 1C, (**b**) E-type curves for SFC No. 2 and 2C, (**c**) E-type curves for SFC No. 3 and 3C, (**d**) E-type curves for SFC No. 4 and 4C, (**e**) F-type curves for SFC No. 1 and 1C, (**f**) F-type curves for SFC No. 2 and 2C, (**g**) F-type curves for SFC No. 3 and 3C, (**h**) F-type curves for SFC No. 4 and 4C.

**Figure 9:**
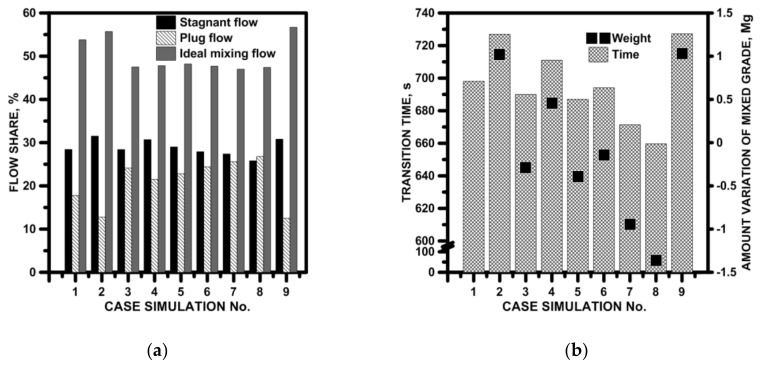
Hydrodynamics in the considered tundish with SFC: (**a**) active flow and stagnant flow, (**b**) transition zone.

In order to assess the influence of the designed flow control device on the hydrodynamic structure forming in the working space, the characteristics of the residence time distribution (RTD) were determined (Figure 8). The qualitative analysis showed that the modification of the working space of the tundish, for both SFCs with, and without, a cavity, did not limit the mass transport processes in the volume of liquid steel in relation to the flow in the bare tundish (the value of the dimensionless marker concentration in the peak of the RTD E curve oscillated close to the value of 0.9). On the other hand, the change in the distance of the E-type RTD peak from the Y axis indicated an increase in the plug flow and a reduction in the stagnant flow. In addition, the presence of bypass flows, as being characteristic of RTD curves with double peaks in the top zone of the curve, was diagnosed (Figure 8a,c). The distribution of the F-type RTD curves confirms increase in the plug flow share in the tundish for SFC No. 2 and 2C as well as 3 and 3C (Figure 8f,g). The F-type RTD curves in these figures are characterized by a greater distance from the *Y*-axis at the down slope of the curve and a smaller distance from the *Y*-axis at the top of the curve. The recorded RTD curves were the basis for quantitative analysis in which the percentages of the stagnant, plug and ideal mixing flows were calculated (Figure 9a). In the bare tundish, the stagnant flow was 28.4%. On the other hand, in the tundish with the SFC without a cavity, no reduction in the share of the stagnant flow was achieved, and only in the case of SFC No. 2 did the share of the stagnant flow remain the same. In the case of using a cavity in the SFC, a slight reduction in the range of stagnant flow was obtained, reaching 2.5% in the case of using SFC No. 3C. Definitely, the SFC influences the increase in the volume of the plug flow share. In the variants of the tundish with SFC No. 2, 3 and 4, an increase in the plug flow volume of 6.6%, 3.8% and 5.1% were obtained, respectively. On the other hand, in the variants of the tundish with SFC 1C, 2C and 3C, plug flow shares increased by 6.7%, 7.9% and 9.1%, respectively. Increasing the plug flow share is important in the case of processes where limiting the mixing intensity is beneficial; for example, when successively casting grades with different chemical compositions, it limits the span of the transition zone. In the bare tundish, the transition zone was 698 s, assuming the transition is in the dimensionless concentration range of 0.2–0.8 [54]. In order to reduce it, it is not enough to increase the plug flow, but also to limit the share of the stagnant flow in the overall hydrodynamic structure. Therefore, only in the tundish with SFC numbers 2, 4, 1C, 2C and 3C was there a reduction in the extent of the transition zone. On the other hand, significant reduction from the point of real continuous steel casting process was achieved only for tundishes with SFC no. 2C and 3C, achieving a decrease in the mass of the steel with an indirect composition of 0.94 Mg and 1.36 Mg, respectively (Figure 9b).

#### 4.2.3. Influence of Internal Working Space of STC

**Figure 10:**
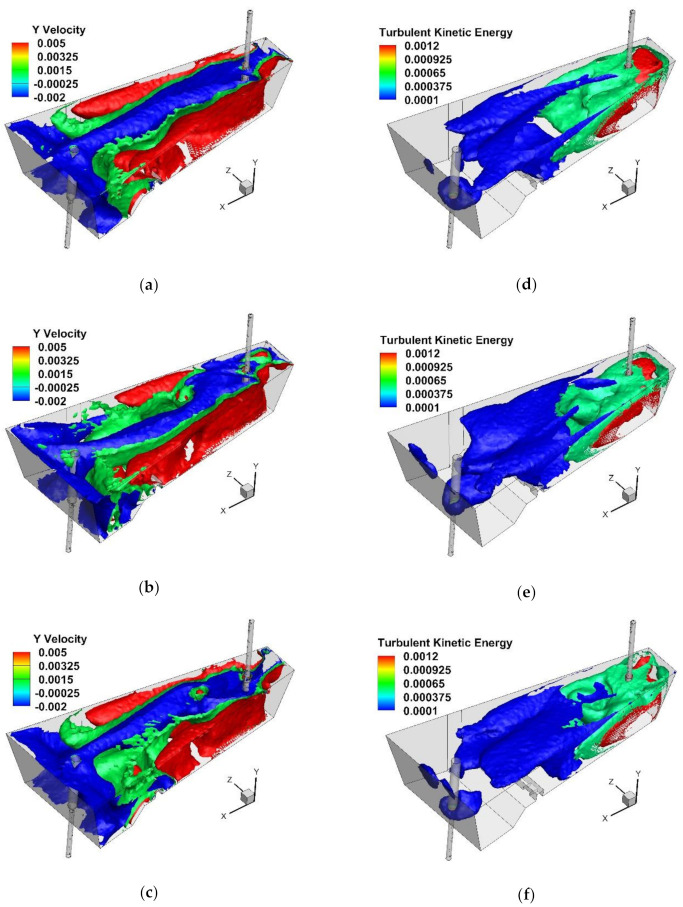
Y component velocity (m/s) and turbulent kinetic energy (m^2^/s^2^): (**a**,**d**) tundish with SFC No. 2C105, (**b**,**e**) tundish with SFC No. 2C125, (**c**,**f**) tundish with SFC No. 2C145.

**Figure 11:**
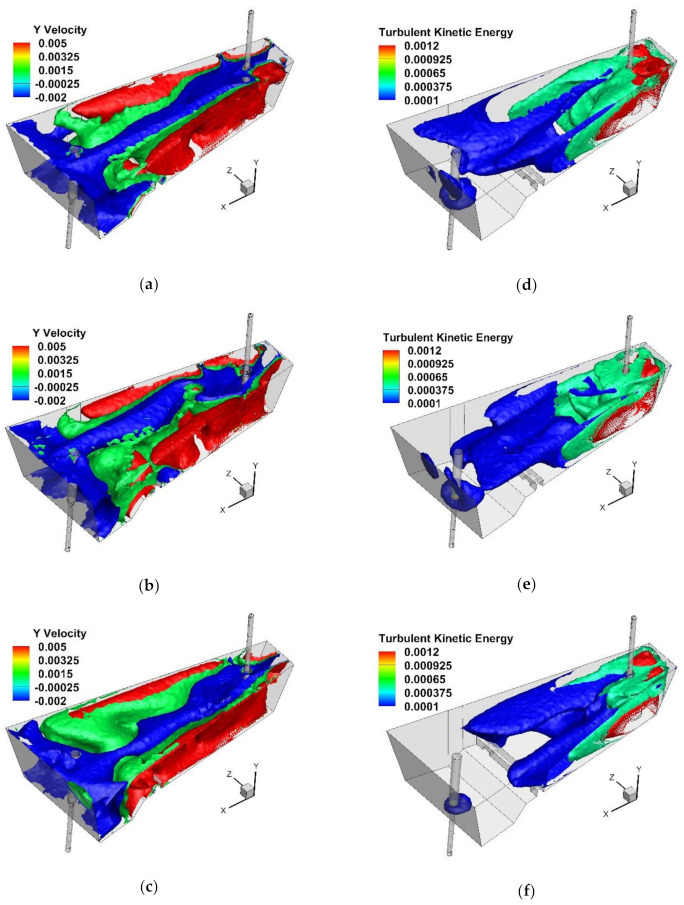
Y component velocity (m/s) and turbulent kinetic energy (m^2^/s^2^): (**a**,**d**) tundish with SFC No. 3C105, (**b**,**e**) tundish with SFC No. 3C125, (**c**,**f**) tundish with SFC No. 3C145.

**Figure 12:**
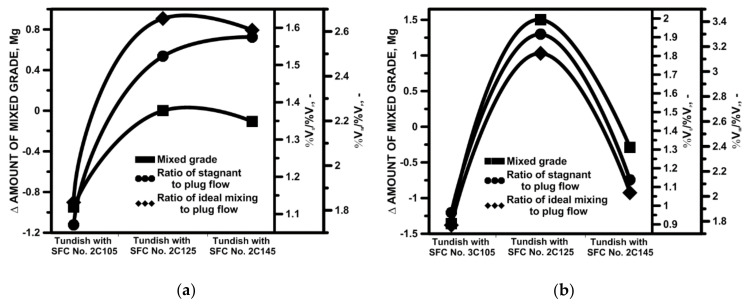
Influence of active flow on transitions grade: (**a**) tundish with SFC No. 2C, (**b**) tundish with SFC No. 3C.

On the basis of the obtained results, it was found that SFC Nos. 2C and 3C have potential for industrial application in the tundish (Figure 10a and Figure 11a). In the next stage of the studies, the influence of changing the cavity diameter by 0.02 m was verified. Both in the case of SFC 2C and 3C, modification of the cavity’s internal space reduced the average flow velocity of the liquid steel in the tundish. The mean flow velocity in the tundish with SFC No. 2C145 and No. 3C145 were 0.0306 m/s and 0.0281 m/s, respectively. In addition, in tundish with SFC 2C or 3C, changing the base surface area of the cavity locally modified the distribution of rising and falling streams. In the central part of the tundish, downstream generally predominates. However, modification of the SFC workspace stimulated small areas of ascending flow in the center of the tundish (Figure 10b,c and Figure 11b,c). A different hydrodynamics occurred in the tundish made of the SFC No. 3C145, where the zone of influence of rising streams in the central part of the tundish increased, which is the effect of slowing down the flow of the liquid steel and an additional influence on the flow of natural convection forces (Figure 11c). The gradient of the temperature and steel density additionally modified the flow of the buoyancy number (Bu), which reached a value above 5 [30,55,56,57]. In this variant of the tundish in the zone of the stopper rod system, the Bu number was 4.57 and was 80% higher than in the bare tundish. In the remaining variants of the tundish with SFC No 2C and No. 3C, and their modifications, the value of the Bu number did not exceed the value of 4. In the base variants of the recessed SFC, the Bu number was 3.14 and 3.25 for SFC no. 2C and SFC no. 3C, respectively. The use of the SFC also influences the mean value of the kinetic energy of the turbulence in the liquid steel, reducing its value by 10% and 13% for SFC No. 2C and SFC No. 3C, respectively (Figure 10d and Figure 11d). On the other hand, the change of the surface area of the cavity base in both SFC variants does not significantly change the value of the average kinetic energy of the turbulence (Figure 10e,f and Figure 11e,f). In Figure 10d–f and Figure 11d–f iso-surfaces of turbulence kinetic energy distribution for the values of 0.0012 and 0.0001 are shown, and the average value for a given variant of the modification of the internal tundish space. Compared to the initial value of the TKE of the feed stream, the level of the TKE reduced by 97% in the tundish’s filling zone. On the other hand, before, and in the stopper rod zone, the kinetic energy of turbulence was 99.5% lower than the value of the stream flowing out of the ladle. The distribution of the kinetic turbulence energy also coincided with the path of the main streams flowing to the tundish stopper rod system. More than a 25% reduction in the average flow velocity in the tundish of SFC No. 3C145 was also reflected in the image of the distribution of the TKEs in the liquid steel volume. The iso-surface for the TKEs equal to 0.0001 m^2^/s^2^ was definitely retracted towards the intermediate tundish pouring zone. The impact of the modification of both SFC variants on the hydrodynamic system in the tundish was verified by determining the RTD curves of types E and F. Figure 12 shows the impact of modifying the SFC cavity on the range of the transition zone as a function of the change in the shares of the individual types of flow. In the base tundish, the stagnant flow to the plug flow and ideal mixing to the plug flow were 1.59 and 3.02, respectively. In the case of the SFC 2C, increasing the area of the base of the cavity did not measurably affect the hydrodynamic system, reducing the range of stagnant flow or modifying the active flow zones. Only in the case of the modification variant SFC No. 3C, with a cavity base diameter of 0.145 m, was a slight improvement in hydrodynamic conditions obtained. In this tundish variant, however, the hydrodynamic system was additionally affected by the forces of natural convection. 

The effectiveness of influencing the steel flow hydrodynamics in the tundish is improved by the use of a cavity in the SFC. On the other hand, increasing the surface area of the cavity base in the tested range did not bring significant additional changes to the structure of the liquid steel flow in the working volume.

#### 4.2.4. Influence of Liquid Steel Pouring Initial Temperature

**Figure 13:**
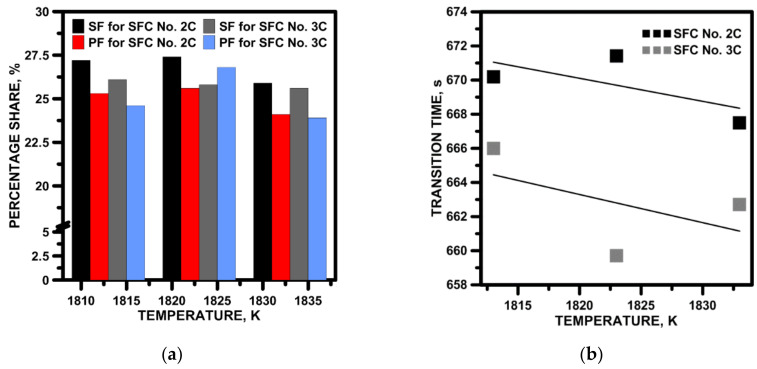
Influence of liquid steel initial temperature: (**a**) influence on stagnant flow (SF) and plug flow (PF), (**b**) influence on transition zone.

During casting sequences, the liquid steel is characterized by a certain level of superheat above the liquid’s temperature, hence it was important to know if temperature change would affect the hydrodynamic system in the designed tundish working space. In this part of the studies, the basic SFC variants 2C and 3C were tested. A change in temperature of ±10 K did not have a significant effect on the stagnant or the plug flow, and nor, thus, on the ideal mixing, in the overall hydrodynamic structure (Figure 13a). This fact was confirmed by the average standard deviation of 0.9%. The stability of the hydrodynamic system, in terms of the influence of temperature, was also confirmed by the span of the transition zone, which, in the case of the SFC No. 2C, changed within ±2 s (Figure 13b). The negligible influence of temperature on steel flow was also the result of the dominance of inertia convection forces over natural convection forces due to the maximum value of the Bu number, equal to 3.14 and 3.48, respectively, in the tundish with SFC No. 2C and No. 3C. 

#### 4.2.5. Influence of Casting Speed

**Figure 14:**
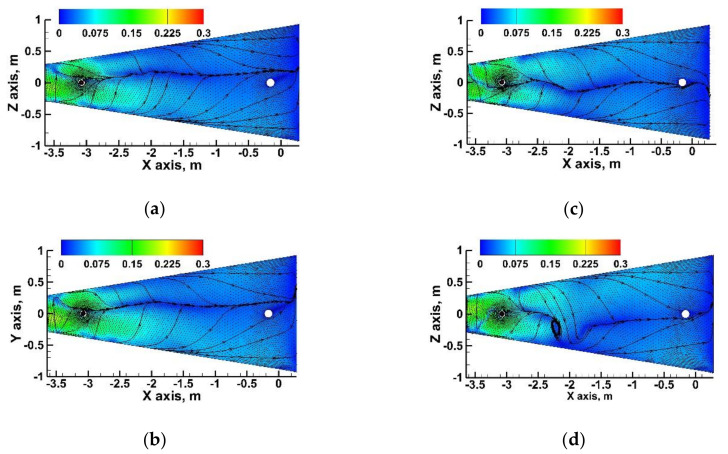
Behavior of liquid steel flow near the tundish flux-steel interface: (**a**) tundish with SFC No. 2C105 and casting speed 1.0 m/min, (**b**) tundish with SFC No. 2C105 and casting speed 1.1 m/min, (**c**) tundish with SFC No. 3C105 and casting speed 1.0 m/min, (**d**) tundish with SFC No. 3C105 and casting speed 1.1 m/min.

**Figure 15:**
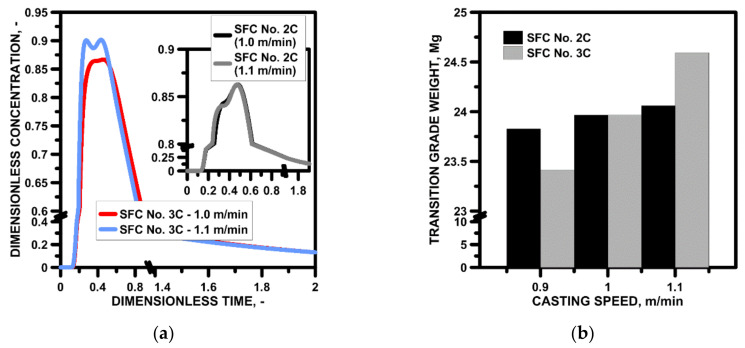
Casting speed: (**a**) influence on stagnant and active flow, (**b**) influence on transition zone.

Slabs of certain grades can be cast at different dedicated speeds, increasing the efficiency of the casting machine. Therefore, the influence of casting speed on the shaping of liquid steel flow structure in tundishes with SFC No. 2C or SFC No. 3C were verified. As the casting speed increased, the driving force of the feed stream increased. Hence, it may be difficult to maintain stability of the steel-slag interface. However, as indicated by the flow maps shown in Figure 14, even in the case of an increase in casting speed by 0.2 m/min in the meniscus zone, the flow velocity of the steel did not exceed the critical speed of 0.3 m/s. The more asymmetric steel flow in the tundish with SFC No. 3C is noteworthy, especially at the casting speed of 1.1 m/min. In addition, in this variant of calculations, there is a characteristic vortex recirculation that can stimulate the formation of a vortex funnel, through which the slag phase will be drawn into the volume of the liquid steel. The hydrodynamic state diagnosed in the upper part of the liquid steel volume, caused by the change of casting speed, is reflected in the general hydrodynamic structure describing the steel flow through the tundish. Figure 15a shows E-type RTD curves. In the case of using SFC No. 2C, the distribution of the RTD curves was very similar; hence, the difference between the stagnant zones for casting speed 1 and 1.1 m/min was 0.1%. On the other hand, in the case of the tundish with SFC No. 3C, increase in casting speed by 0.2 m/min definitely affected the hydrodynamic structure in which the bypass flow appeared, described by two peaks in the top part of the curve. The share of stagnant flow increased slightly (2%) and the share of plug flow decreased in relation to casting at a speed of 0.9 m/min via tundish with SFC No. 3C. Hence, the weight of the steel in the transition zone increased as casting speed grew. On the other hand, the stabilization of the hydrodynamic conditions in the tundish equipped with SFC No. 2C in the tested casting speed range was also reflected in the amount of indirect composition steel, and differences obtained at the level of 0.25 Mg (Figure 15b). 

#### 4.2.6. Non-Metallic Inclusions Behavior and Tundish Lining Wear

**Figure 16:**
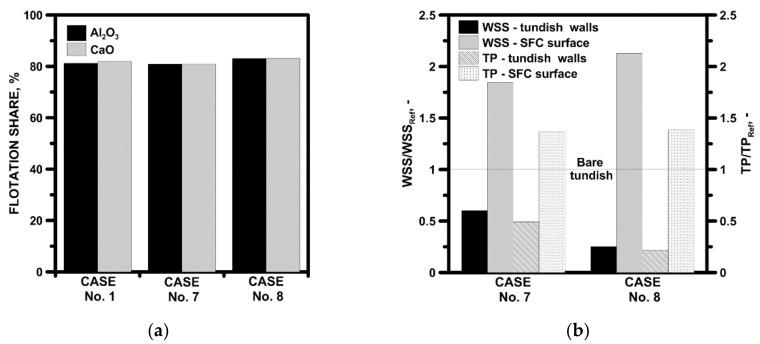
Behavior of non-metallic inclusions and potential of tundish lining wear: (**a**) flotation of non-metallic inclusions, (**b**) wall shear stress and total pressure in tundish and SFC walls.

In the last stage of the studies, the influence of the applied FCDs on the behavior of non-metallic inclusions in the volume of the liquid steel and the working layer of the refractory lining of the tundish and SFC were assessed. For NMIs analysis, Al_2_O_3_ and CaO were selected, which, in industrial conditions, are present in the form of solids. In addition, the specific density of calcium oxide is close to the slightly modified NMIs type Al_2_O_3_⋅CaO. Figure 16a presents the result of the numerical simulation of the NMIs’ flotation to the free surface of the steel. The designed flow control devices did not worsen the conditions for the NMIs’ flotation from liquid steel, keeping it at a level of 81%. Also, 18.5% increase in specific density of the NMI did not reduce NMIs’ flotation potential from liquid steel in the tundish, which is a characteristic for small precipitates. Both SFC No. 2C and 3C did not worsen conditions for the flotation of non-metallic inclusions during the continuous casting process. Figure 16b shows the influence of the tundish equipment on the maximum absolute value of the wall shear stress (WSS) and total pressure (TP), calculated for the walls and bottom of the tundish to the low dam location zone, and, separately, for the external surfaces of the SFC. The WSS and TP reference value is related to the maximum value calculated for the bare tundish and is level 1 in Figure 16b. In the case of using the SFC, there was a significant reduction in the maximum value of the WSS by 40% and 75%, respectively, for SFC 2C and 3C. In addition, a 50% and 80% reduction in the TP value on the walls and bottom of the tundish in the tundish pouring zone confirmed the limitation of the wear conditions of the working layer of the refractory lining of the tundish in its filling zone. The enclosure of the SFC tundish causes the driving force of the steel flowing from the ladle and the erosive effect of the feed stream to be transferred to the SFC. Hence, for SFC No. 2C and No. 3C, respectively, on the outer surface of the SFC, there is more than an 80% and 100% increase in the maximum value of the WSS located mainly at the edges joining the cavity with the surface of the hemisphere. For both SFCs, there is a 35% increase in the maximum TP value. Therefore, the flow control device installed in the pouring zone of the tundish is particularly exposed to the erosive influence of the steel feed stream and, in order to fulfill its function as a flow regulator and refractory lining protector of the tundish, it should be made of materials with an increased service life. In the case of the analyzed flow control devices, SFC No. 2C was more favorable, which, due to its smaller dimensions, can be made of more advanced and more expensive refractory materials. 

## 5. Conclusions

On the basis of computer simulations and physical experiments on the water model, it was found that: 

The provision of a cylindrical cavity in the SFC limits the extent of the stagnant flow zone with respect to the bare tundish. Therefore, with simultaneous increase in plug flow share, the span of the transition zone and the amount of steel with an indirect composition reduced by 0.94 Mg and 1.36 Mg, respectively, when using SFC No. 2C or No. 3C. 

Both SFC No. 2C and No. 3C were characterized by stability of the influence on hydrodynamic structure in the tundish when changing the superheat temperature of the liquid steel in the temperature range of 1813-1833 K. 

In the case of changing the casting speed within the speed range of 0.9-1.1 m/min, SFC No. 2C was more stable, in terms of formation of a hydrodynamic structure. 

SFC no. 2C and No. 3C limit the erosive effect of molten steel on the refractory lining of the tundish. However, considering the influence of the SFC on the steel flow structure in the tundish and the gradient of the WSS and TP on the SFC surface, the most optimal SFC variant for a single-strand wedge-type tundish is SFC No. 2C.

## Figures and Tables

**Table 1:** Considered variants of tundish equipment and casting conditions.

Case No.	BT	Type of FCD											Inlet Temperature, K			Casting Speed, m/min		
		Hemisphere SFC				Hemisphere SFC with Cavity				Diameter of Cavity, mm								
		1	2	3	4	1C	2C	3C	4C	105	125	145	1823	1813	1833	0.9	1.0	1.1
1 2 3 4 5 6 7 8 9 10 11 12 13 14 15 16 17 18 19 20 21	x - - - - - - - - - - - - - - - - - - - -	- x - - - - - - - - - - - - - - - - - - -	- - x - - - - - - - - - - - - - - - - - -	- - - x - - - - - - - - - - - - - - - - -	- - - - x - - - - - - - - - - - - - - - -	- - - - - x - - - - - - - - - - - - - - -	- - - - - - x - - x x - - x x - - x x - -	- - - - - - - x - - - x x - - x x - - x x	- - - - - - - - x - - - - - - - - - - - -	- - - - - x x x x - - - - x x x x x x x x	- - - - - - - - - x - x - - - - - - - - -	- - - - - - - - - - x - x - - - - - - - -	x x x x x x x x x x x x x - - - - x x x x	- - - - - - - - - - - - - x - x - - - - -	- - - - - - - - - - - - - - x - x - - - -	x x x x x x x x x x x x x x x x x - - - -	- - - - - - - - - - - - - - - - - x - x -	- - - - - - - - - - - - - - - - - - x - x

## Data Availability

Not applicable.
